# Laparoscopic reoperation of the bile duct in a patient with situs inversus totalis: a case report

**DOI:** 10.1093/jscr/rjae671

**Published:** 2024-11-01

**Authors:** Shengchang Zhu, Liang Luo

**Affiliations:** Department of Hepatobiliary Surgery, YiChun City People's Hospital, 1061 Jinxiu Avenue, Yichun 336000, Jiangxi, China; Department of Hepatobiliary Surgery, YiChun City People's Hospital, 1061 Jinxiu Avenue, Yichun 336000, Jiangxi, China

**Keywords:** situs inversus totalis, laparoscopic surgery, bile duct reoperation

## Abstract

Situs inversus totalis is a rare congenital anomaly, where the positions of major internal organs are reversed from their normal arrangement. This anatomical abnormality significantly increases the complexity of surgeries, especially in laparoscopic reoperations of the bile duct for patients with a history of abdominal surgery. Traditional anatomical landmarks and surgical steps need to be adjusted according to the patient’s unique anatomy. This not only makes intraoperative identification of structures more challenging but also introduces greater uncertainty during the procedure. This case report summarizes the successful laparoscopic reoperation of the bile duct in a patient with situs inversus totalis, providing important reference and guidance for future similar procedures.

## Introduction

Situs inversus totalis (SIT) is a rare congenital anomaly characterized by the mirror-image arrangement of internal organs compared to the normal anatomical layout [1]. The incidence is approximately 1 in 10 000 to 50 000 live births [2]. Although most individuals with SIT are asymptomatic and may remain undiagnosed throughout life, this condition presents significant challenges when surgery is required. Particularly in abdominal surgeries, traditional anatomical landmarks and surgical techniques must be adjusted to accommodate the patient’s reversed anatomy. This requires a surgical team with extensive experience and thorough preoperative imaging assessments to ensure the success of the surgery. While bile duct stone recurrence is not uncommon after cholecystectomy, cases with concurrent SIT are extremely rare. The abnormal arrangement of organs in such cases increases the difficulty of the surgery. This case details the successful laparoscopic bile duct reoperation for a patient with SIT, performed at our department in July 2024, and summarizes our experience.

## Case presentation

**Figure 1:**
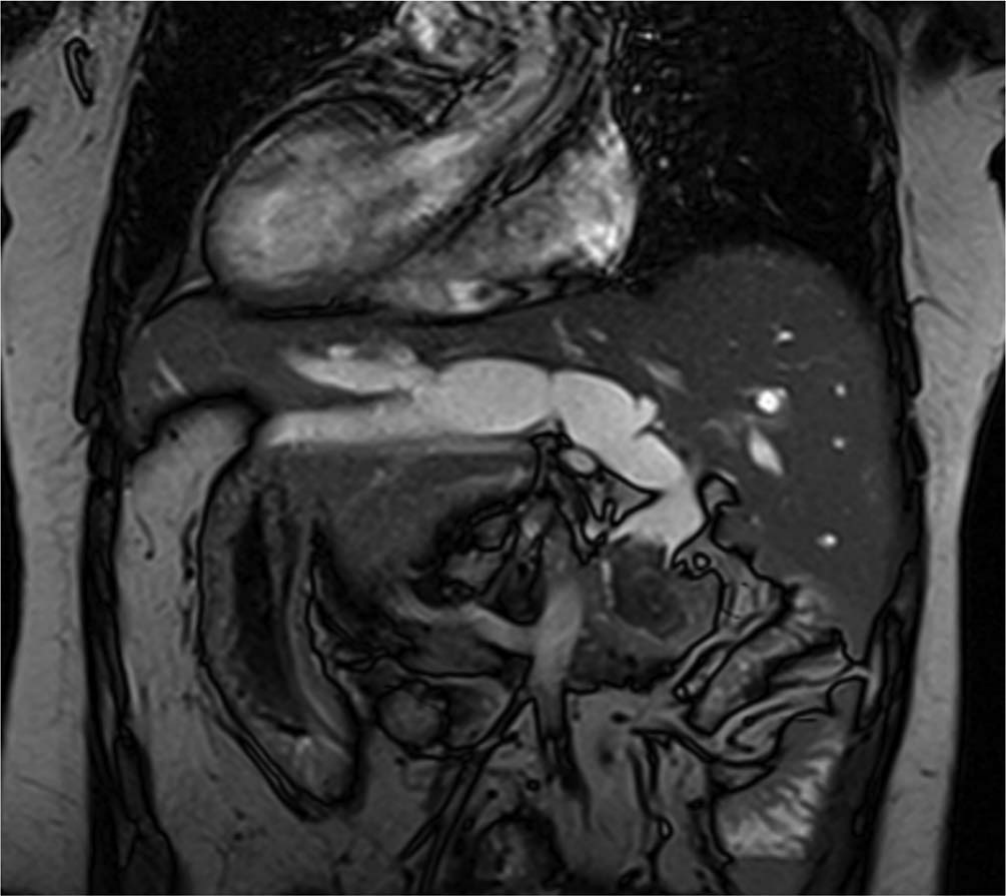
MRI shows a 26 mm stone in the distal common bile duct with bile duct dilation.

**Figure 2:**
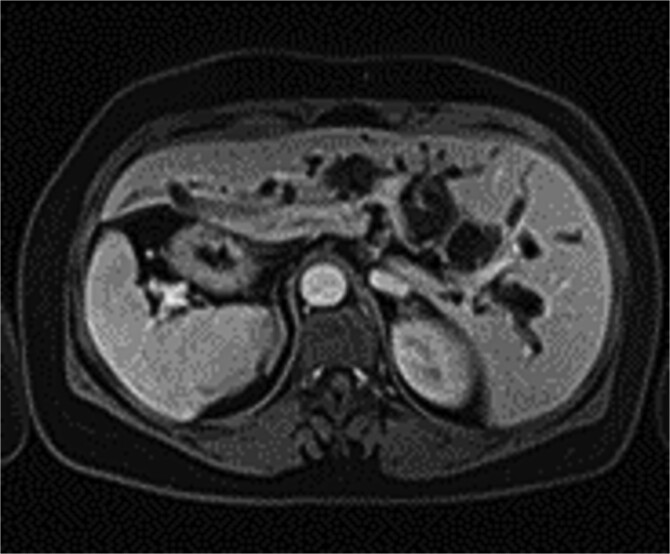
Abdominal organs in mirror-image arrangement.

The patient, a 56-year-old female, was admitted on July 7, 2024, with a one-day history of abdominal pain and fever. The pain, which began on July 6 after consuming greasy food, was paroxysmal and located in the upper abdomen, accompanied by chills and a fever as high as 39°C. CT imaging revealed common bile duct stones and post-cholecystectomy status. Physical examination upon admission showed jaundice, with mild tenderness in the left upper quadrant, without rebound tenderness, and a negative Murphy’s sign. MRI and MRCP of the upper abdomen revealed bile duct dilatation due to a 26 mm stone in the distal common bile duct, with mirror-image arrangement of the internal organs (Figs. 1 and 2). The diagnosis was choledocholithiasis with cholangitis and situs inversus totalis. After completing preoperative evaluations, the patient underwent laparoscopic common bile duct exploration, choledochotomy for stone extraction, choledochoscopy, T-tube drainage, and lysis of adhesions under general anesthesia on July 9, 2024.

**Figure 3:**
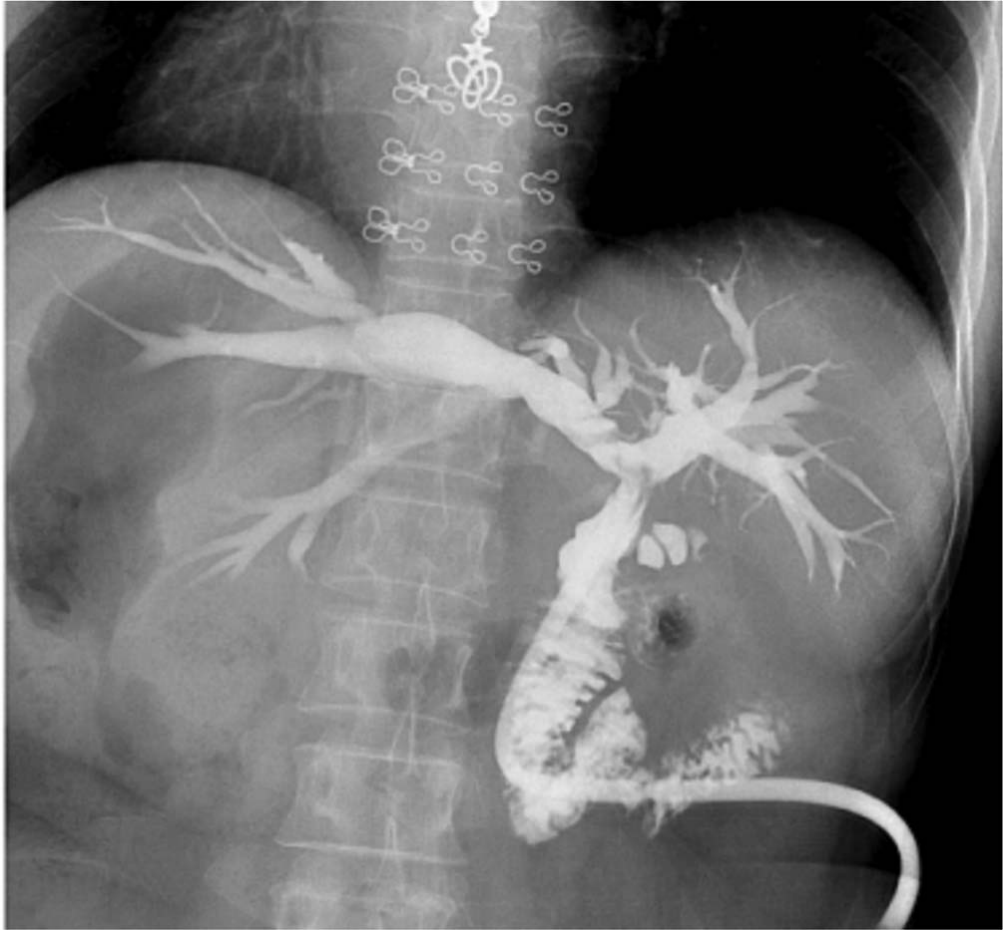
Postoperative cholangiography shows no residual stones in the bile duct.

Intraoperatively, the liver was located on the left side, with the spleen and stomach on the right. The surgeon stood on the patient’s right side, with the patient in a reverse Trendelenburg position (30° head-up, 15° left-side down). Adhesions between the liver, omentum, and surrounding organs were carefully dissected to expose the common bile duct, which measured 2.0 cm in diameter. A 1.5 cm longitudinal incision was made on the common bile duct, and stones were extracted using atraumatic forceps and a stone retrieval basket. Choledochoscopy confirmed no residual stones in the intrahepatic or extrahepatic bile ducts. Due to marked edema at the lower end of the common bile duct caused by stone impaction, a 22 French T-tube was placed. The procedure lasted 125 minutes, with no postoperative bile leakage or other complications. The patient had an uneventful recovery and was discharged on postoperative day 5. Two weeks later, cholangiography showed no residual stones in the bile duct (Fig. 3).

## Discussion

Situs inversus totalis is a rare condition that presents significant challenges when surgical intervention is required due to the mirror-image arrangement of organs and the absence of standardized surgical approaches. Most reported laparoscopic surgeries in SIT patients have involved initial operations, with very few cases of laparoscopic reoperations for the bile duct.

**Figure 4:**
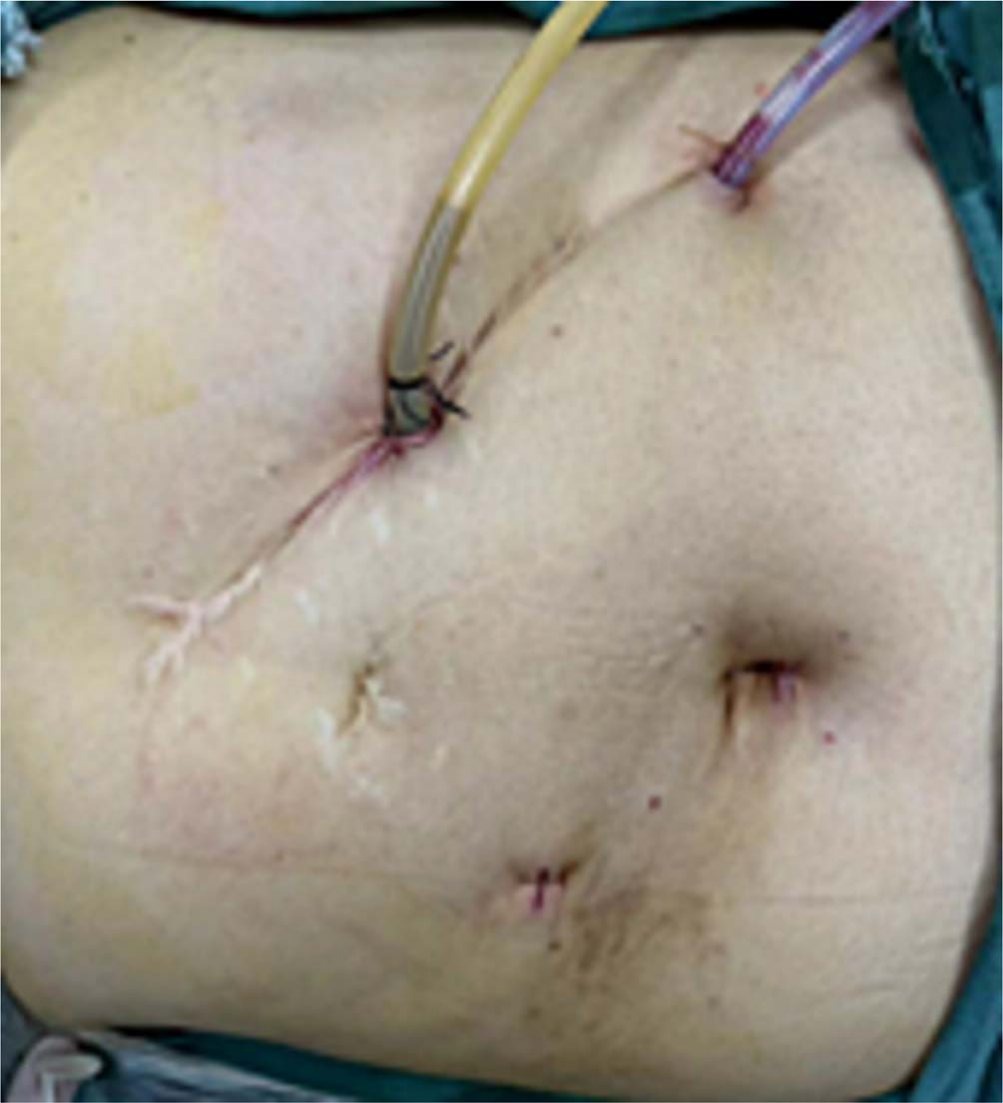
Postoperative condition of the patient’s abdominal incision.

There is no consensus on the optimal positioning of the surgeon for SIT patients. Kigasawa *et al. * [3] successfully performed surgery with the lead surgeon standing on the right and the assistant on the left in a reversed position. Yaegashi *et al. *[4] also reported similar success with this approach, emphasizing the importance of adjusting the surgical team’s positions for SIT patients. In our case, severe adhesions in the upper abdomen, particularly on the left side, led us to select the right midclavicular line, 3 cm above the umbilicus, as the main working port. The original incision along the left midclavicular line under the costal margin was used as the secondary working port for T-tube placement and external drainage, while an additional port was placed along the left anterior axillary line for inserting the abdominal drain (Fig. 4). The surgical team employed a reversed setup, which facilitated the successful completion of the procedure.

Cases of SIT with recurrent bile duct stones are rarely reported, and in this patient, the severe adhesions from previous bile duct surgeries further complicated the anatomy and increased the difficulty and risk of the procedure. A thorough preoperative imaging evaluation is critical, not only to identify the reversed organs but also to check for any vascular, biliary, or intestinal abnormalities. Intraoperatively, precise identification of the bile ducts and blood vessels at the hepatic hilum is essential. Blind clamping or cutting of tubular structures must be avoided, and in cases of severe adhesions or difficult dissection, timely conversion to open surgery should be considered [5]. We conclude that laparoscopic bile duct reoperation in patients with SIT is safe and feasible, but the reversed anatomy and severe adhesions require highly skilled laparoscopic surgeons. The main challenges lie in the surgeon’s ability to adapt to reversed visual and spatial cues, and careful preoperative planning and intraoperative adjustments are crucial to the success of the surgery. This case provides valuable insights and experience for performing laparoscopic reoperations in SIT patients with recurrent bile duct stones.

## Conclusion

Reoperation on the bile duct in patients with situs inversus totalis poses significant challenges. However, with thorough preoperative evaluation and careful intraoperative techniques, favorable outcomes can be achieved. This case report highlights important surgical strategies and provides valuable experience for future similar procedures.
